# Defect phases beyond grain boundaries

**DOI:** 10.1557/s43577-025-01044-0

**Published:** 2026-02-17

**Authors:** Sandra Korte-Kerzel, Timothy J. Rupert, Daniel S. Gianola, Stefanie Sandlöbes-Haut, Zhuocheng Xie

**Affiliations:** 1 Institute for Physical Metallurgy and Materials Physics, RWTH Aachen University, 52074 Aachen, Germany; 2 Hopkins Extreme Materials Institute, Johns Hopkins University, Baltimore, USA; 3 Materials Department, University of California, Santa Barbara, Santa Barbara, USA

**Keywords:** crystal, alloy, defects, dislocations, thermodynamics

## Abstract

Defects are fundamental to the behavior and performance of structural materials, yet their treatment in alloy design has often been decoupled from thermodynamic considerations of phase stability. The emerging concept of "defect phases" — chemically and structurally distinct configurations at lattice defects — offers a unified framework that integrates defect chemistry, thermodynamic stability, and mechanical behavior. While grain-boundary (two-dimensional) defect phases have gained recent attention, this article expands the scope to include defect phases across all dimensionalities, with a particular emphasis on dislocations (one-dimensional) as mobile carriers of plastic deformation and sites of complex phase behavior. We discuss how point, line, and planar defects can host distinct defect phases, how these phases compete for solute atoms, and how their stability can be mapped using defect phase diagrams constructed in chemical potential space. Through selected case studies in metallic solid solutions and ordered intermetallics, including Laves, B2, and µ-phases, we illustrate how dislocation-based defect phases can influence plasticity, strengthen alloys, or even drive local transformations that modify mechanical properties. By bridging defect physics with materials thermodynamics, we advocate for a defect phase-informed design paradigm that connects atomic-scale phenomena to bulk processing and performance.

**Graphical abstract:**

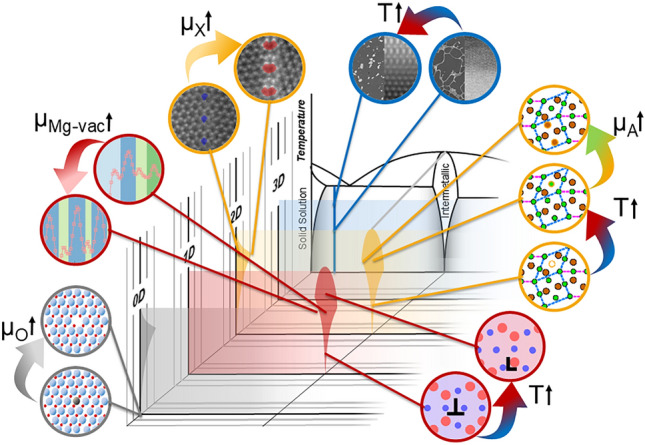

## Introduction to defect phases across dimensionalities

The performance and reliability of structural materials are intrinsically linked to the presence and behavior of defects within their crystal lattices. Defects such as vacancies, solutes, dislocations, grain boundaries, stacking faults, and phase boundaries significantly influence mechanical properties, corrosion resistance, and other critical material characteristics. Traditionally, two approaches have proven immensely successful in alloy design: (1) thermodynamic and kinetic descriptions for tailoring and processing alloys to achieve desired microstructures; and (2) manipulation of crystal defects to control properties such as strength and formability. However, these concepts have largely remained decoupled.

**Figure 1:**
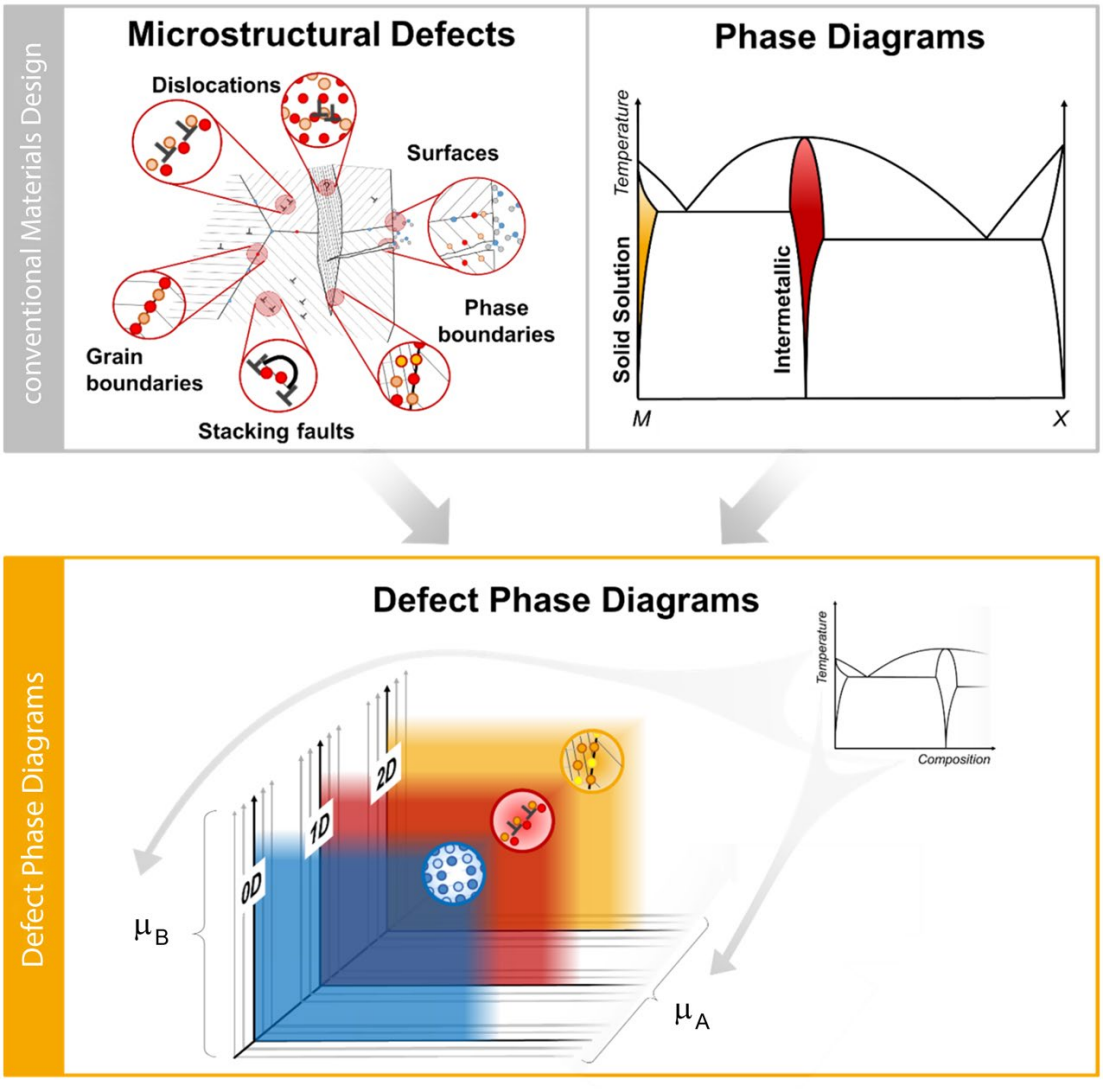
Framework for considering defect phases of different dimensionalities together for materials design. Reprinted from Reference 1, published under a CC-BY 4.0 license.

A bridge is needed between these powerful approaches to achieve a single conceptual framework, as illustrated in **Figure** 1. Considering defects and their thermodynamic state holistically as defect phases provides a future materials design strategy by jointly treating the thermodynamic stability of both the local crystalline structure and the distribution of elements at defects.

A “defect phase” has been defined by Korte-Kerzel et al.^1^ as “a structurally and chemically distinct atomic-scale defect configuration for which all physical properties can be expressed as smooth (continuous and infinitely differentiable) functions of intensive control variables such as temperature or chemical potential.[...] A defect phase can only exist in the vicinity of its defect.” This has major implications, most importantly that by considering the possible states of defects in terms of both structure and chemistry at the atomic scale, one can predict the energetically most favorable defect phases and consider their competition across all dimensionalities (point, line, planar defect) within different bulk phases including their own atomic order where applicable.^1^

Experimental and theoretical studies suggest that defect phases are indeed ubiquitously present and can undergo transitions analogous to bulk phases with a variety of highly ordered local structural and thermodynamic defect states.^2–10^

In this article, we expand upon the theme of grain-boundary defect phases considered in this issue and introduce the concept of defect phases irrespective of their dimensionality. We focus in our examples on one-dimensional (1D) defects (dislocations) as the carriers of plastic deformation and how their states and transitions are related to properties of structural as well as functional materials. We consider cases where the matrix phase is a solid solution and the defect phases can establish chemical ordering, as well as instances where the matrix phase is an ordered intermetallic, and explore commonalities.

## Defect phases across dimensionalities

Defect phases can exist in various types of lattice defects, including point defects (zero-dimensional [0D]), dislocations (1D), grain or phase boundaries, and stacking faults (two-dimensional [2D]). Understanding defect phases across all dimensionalities is crucial for a comprehensive picture of microstructural evolution and materials properties. This must include not only the properties resulting from each potential defect phase, but also their properties as an ensemble and their competition for solutes in the matrix they share.

**Figure 2:**
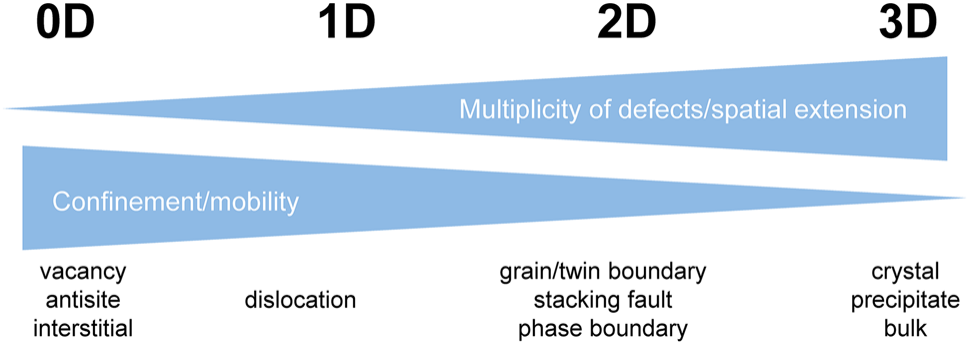
Multiplicity and extension of defects versus their mobility and confinement.

Importantly, the dimensionality of a defect is intricately related to the multiplicity of defect phases that may be formed, but also to fundamental properties dominated by each type of defect (**Figure** 2).

A natural hierarchy exists, where the simplest 0D defects may be contained or interact with 1D or 2D defects. Similarly, dislocations as 1D defects are an integral part of many grain boundaries or phase boundaries and may bound stacking faults. At the same time, the spatial extension of the defects increases with their multiplicity, ranging from a point defect with an extending strain field affecting a few neighboring atoms to planar boundaries spanning across entire macroscopic samples (while their third dimension normal to the defect plane remains constrained). These defects may reversely be considered increasingly confined with decreasing dimensionality, which leads to increased mobility. Where point defects, particularly vacancies, enable diffusion, dislocations may move readily under applied stresses. Together, they also relieve stresses by rapid diffusion along the dislocation core. Two-dimensional boundaries in turn depend on dislocations to provide sufficient driving force for their motion, enabling microstructural evolution during recrystallization or stimulation of precipitation.

The 1D line defects are therefore the central defect balancing mobility with spatial confinement. As such, they have the ability to form a multitude of defect phases with distinct atomic and chemical structures and can also be highly mobile. The latter relates them to accessible materials properties, most prominently during plastic deformation in the form of active slip systems and critical stresses for their activation. For instance, while grain boundaries and their local states may indirectly influence the progression of plastic deformation and mediate strength, the dislocations themselves are the vehicle for plastic slip. Targeting defect phases within the dislocation microenvironment, thus, promises a direct knob for controlling properties. This viewpoint contrasts with the conventional approach of decorating a microstructure with obstacles for dislocation motion while assuming the mobile dislocations to maintain constant structure and properties. In the defect phase framework, the features otherwise thought to be obstacles for dislocation motion are spatially co-located as features that modify the dislocation microenvironment.

In order to highlight the connection between defect phases, how to consider their competition and stability, and linking defect phases with materials properties, we focus here on dislocations as the central defects in our examples, touching upon 0D and 2D defects in their interactions.

## Defect phase diagrams in materials design

**Figure 3:**
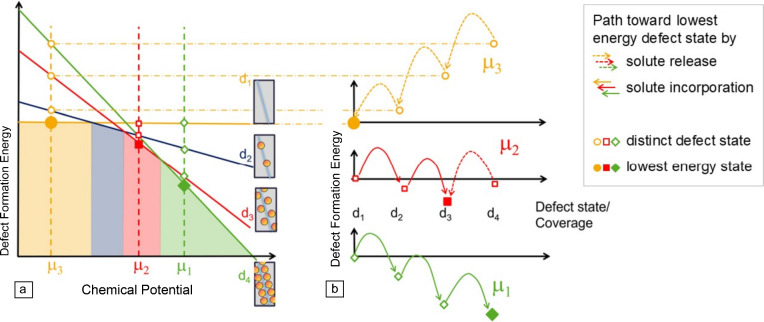
Connection between a metastable defect phase diagram in (a) and the underlying defect state transitions in (b). The defect formation energy, chemical potential µ, and the available defect states form the three dimensions connected in (a) and (b). Axes, and illustrations of the most stable defect states (defect phases). Reprinted and adapted from Reference 11, published under a CC-BY license.

To address defects of all dimensionalities in one framework, defect phase diagrams were proposed as an extension of bulk phase diagrams in extension of early work on surfaces and grain boundaries and considering our more recent abilities to study defects at atomic resolution in both experiments and computational modeling. Similar to bulk phase diagrams considering all possible stable phases, defect phase diagrams map the (meta)stability regions of different defect phases as a function of thermodynamic variables, for example, the chemical potential (**Figure** 3). They extend the traditional bulk phase diagrams by incorporating the dimensionality and unique characteristics of defects. Replacing concentration as the thermodynamic state variable in bulk diagrams with the chemical potential in defect phase diagrams overcomes many limitations in their construction. In thermodynamic equilibrium, the chemical potential is constant and identical at the defect, in the bulk, and in the environment.^12,13^ This provides a convenient and direct comparison between various defect types, the bulk, and the environment. Using chemical potentials allows for the consideration of local variations in composition while maintaining a consistent thermodynamic framework. It also simplifies the integration of defect phase diagrams with mechanism property diagrams and engineering parameters, facilitating materials design.^1^

For the design of materials, defects of all dimensionalities are of importance. Point defects may provide conductivity or play an important role in their interaction with higher dimensional defects, such as in solid-solution hardening observed in metals or vacancy softening proposed in intermetallics.^14^ At dislocations, segregation may take the form of specific structures with chemical order. The structure and chemistry of dislocation cores in turn significantly influence their mobility, affecting macroscopic mechanical properties, such as yield strength and ductility.^4,5^ Dislocations further interact with grain boundaries, phase boundaries, may bound stacking faults, and exist within a lattice that may be effectively solute-free, contain disordered solute atoms, short-range ordered clusters, or even a fully ordered lattice.

**Figure 4:**
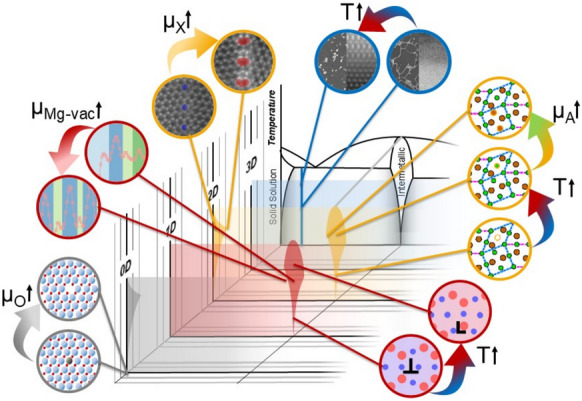
Examples of defect phases and their transitions in a framework considering defect phases and their (meta)stability analogously to bulk phase diagrams. Here, the respective bulk phase is indicated as well as the dimensionality of the considered defect. Defect phase diagrams form the basis of the underlying transitions studied in detail in References 14–18.

Understanding the formation, competition, and properties of defect phases forms the basis for a purposeful manipulation of segregation using thermodynamic driving forces, which allow the translation of these atomic-scale phenomena to bulk processing of mass-produced engineering alloys. This can be achieved by integrating defect phases and their diagrams into materials design, and developing a unified framework that simultaneously considers bulk phases and defects as proposed by Korte-Kerzel et al.^1^ and shown schematically in Figure 1 and for specific examples of 0D, 1D, and 2D defect phases and examples of their transitions in **Figure** 4. In such a framework, defect phase diagrams must then be linked to mechanism-property diagrams through common thermodynamic variables, such as the chemical potential.

More detail on how to assess stability of defect states at the atomic scale within crystals and at their surfaces is abundant in the literature,^11,19–21^ with a short historical perspective ranging from surfaces,^22^ Cottrell atmospheres,^23^ and amorphous grain-boundary phases^24^ to today’s atomically resolved structure and chemistry included in References 1 and 25. This issue also contains several contributions considering 2D defects in great detail. Because the thermodynamic approach is transferable also to dislocations, we focus here on dislocation defect phases and how they affect materials properties in metals and intermetallics (i.e., in crystals with and without intrinsic order in their crystal lattice as the host to defect phases).

## Dislocation defect phases in metallic solid solutions

**Figure 5:**
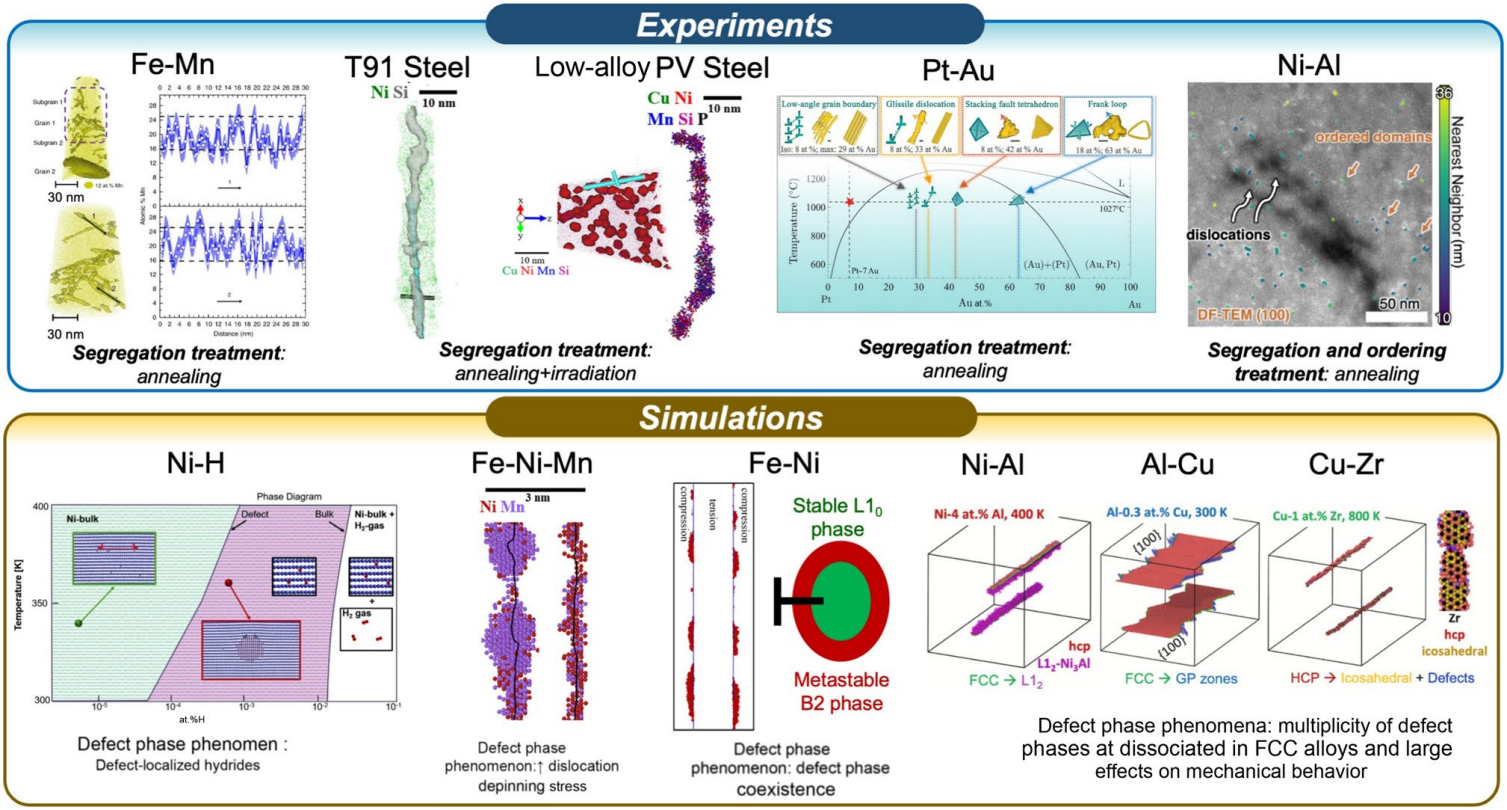
Observations of 1D defect phases in metallic solid solutions. Experimental evidence includes compositional enrichment following segregation annealing of dislocations in Fe–Mn^4,5,35,36^ (reprinted from Reference 36 under a Creative Commons Attribution 4.0 International License) and Pt–Au^39^ (reprinted from Reference 39 under a Creative Commons Attribution Non-Commercial License 4.0), following irradiation and annealing of T91 steel^40^ and a reactor pressure vessel steel,^37,41^ and chemical ordering after heat treatments of dislocations in Ni–Al.^19^ Atomistic simulations of 1D defect phases in Ni–H^33,34^ (reprinted from Reference 1 under a Creative Commons CC BY license), Fe–Ni–Mn,^42^ Fe–Ni,^43,44^ and a range of face-centered-cubic (fcc) alloys with dissociated dislocations hosting partial dislocations with edge components.^45–48^ The T91 Steel and PV Steel images are reprinted and slightly adapted with permission from Reference 37, with permission from ©2021 Elsevier. The Ni–Al experimental image is reprinted with permission from Reference 19. with permission from ©2025 Elsevier. The Fe–Ni–Mn images are reprinted and slightly adapted with permission from Reference 42. with permission from ©2019 Elsevier. The Fe–Ni images are reprinted and slightly adapted with permission from Reference 43. with permission from ©2018 Elsevier. The Ni–Al, Al–Cu, and Cu–Zr simulation images are reprinted and slightly adapted with permission from Reference 46. with permission from ©2023 Elsevier. DF-TEM, dark field TEM; GP zones, Guinier-Preston zones; hcp, hexagonal close-packed.

The concept of dislocations serving as collection sites for solute atoms in metals begins with the discovery of Cottrell atmospheres in 1949 by Cottrell and Bilby,^23^ with interstitial impurites such as C, B, or N acting to pin dislocations. The lattice strain brought about by these impurity atoms can relieve the stresses associated with the dislocation, lowering the defect energy and providing a driving force for segregation. Such features can cause upper yield point phenomena, while also adding viscous drag for dislocations under creep conditions.^26^ Importantly, Cottrell atmospheres could be considered as the first observation of a dislocation defect phase, as the elevated solute concentrations differ from options available on the bulk phase diagram. Suzuki segregation where substitutional solutes enrich stacking faults^27^ would be another early example, with both this defect state and Cottrell atmospheres being chemically disordered examples of defect phases. Both are segregation phenomena that could exhibit well-defined transition temperatures and compositions, analogous to segregation transitions in grain boundaries.^28–30^ Kirchheim^31,32^ proposed the idea of describing dislocation-induced segregation and phase precipitation in Pd-H with a thermodynamic state function that captures the connection between dislocation energy and the chemical potential of the segregating solute, calling these features defactants in an analogy to surfactants. An example of an atomistic simulation of a defactant in Ni–H is shown in **Figure** 5, where nucleation of a localized hydride phase occurs.^33,34^

The first detailed observation of a dislocation defect phase occurred in Fe-Mn by Kuzmina et al.,^5^ where a face-centered cubic (fcc) austenite phase was observed in the body-centered-cubic (bcc) matrix after heat treatment. The local composition along the dislocations matched the bulk austenite concentration, meaning that the defect phase was chemically similar to bulk phase options. However, the defect phase was inherently different structurally, as it was confined to a small region near the dislocation where the stresses drove segregation. Moreover, a repeating array of enriched particles separated by regions of lower concentration were formed as to resemble a pearl necklace pattern. Notably, a structural signature of the dislocation phase could only be captured by diffraction of a low-angle boundary containing many edge dislocations. The need for an edge dislocation confirms that the hydrostatic stress component was responsible for the Mn enrichment, with thermodynamic analysis showing that compressive stress fields stabilized the defect phases.^5,35^ Linear spinodal fluctuations were observed along the dislocation lines in this alloy, with complementary thermodynamic calculations suggesting magnetic ordering as a possible driving force.^36^ Similar dislocation defect phases were reported to form under a combination of annealing and irradiation by Odette et al.^37^ for various reactor pressure vessel steels, showing that multiple driving forces can be active for defect phase nucleation. While these observations in bcc alloys were very important, only modest changes to mechanical properties (e.g., a static strain aging effect in Reference 4 ) were observed due to the formation of defect phases only along a subset of the dislocation network (edge dislocation segments). Atomistic simulations on dislocation defect phases in Fe–Ni showed that such an initial yield point results from a diffusionless and lattice distortive transformation from L1_0_ to B2 once the dislocation breaks away under loading.^38^

The restriction of defect phase observations to edge dislocations in the bcc Fe–Mn example above occurs because only these dislocations have nonzero hydrostatic stress, a key aspect needed to drive segregation of substitutional solutes. In contrast, the dislocations in close-packed crystal structures such as fcc and hexagonal close-packed (hcp) provide a more consistent template for dislocation defect phase formation, as dislocations dissociate into Shockley partials with mixed character bounding an intervening stacking fault. This means that all dislocations will have at least some edge character and associated hydrostatic stress needed to drive substitutional solute segregation and subsequent dislocation defect phase formation. The right side of Figure 5 shows different classes of dislocation defect phases in fcc alloys. The simplest observation of chemical heterogeneity and localized structure formation at a dislocation is an array of nanoparticles that decorate the dislocation line, where local stresses are significantly relaxed yet the dislocation core is unaltered, the defect remains straight, and the very localized structures that form remain confined to the defect. Here, nanoscale regions form that have compositions similar to a bulk phase, yet are structurally distinct because they form a pattern of repeating nanoparticles along the dislocation line as local stresses are relaxed. This type of defect phase is similar to those observed in bcc alloys, in terms of confinement and the pearl necklace pattern. It is important to note that all of these dislocation defect phase particles differ from bulk precipitates that heterogeneously nucleate at a dislocation. Precipitates created by heterogeneous nucleation are not confined to the region near the defect, growing into the bulk and subsequently following traditional ripening laws. Dislocation defect phases do not grow beyond the region near the dislocation where local stresses are significantly elevated. Zhou et al.^39^ observed segregation levels that were specific to the type of dislocation defect (i.e., glissile dislocation, low-angle boundary, stacking-fault tetrahedron or Frank loop) in a Pt-Au alloy, which they hypothesized was due to the different local distortion fields. Howard et al.^19^ demonstrated in Ni–Al that these defect phases were much more commonly observed and produced a more pronounced strengthening effect than their bcc counterparts. These authors showed the first direct evidence of both chemical and structural ordering near dislocations in fcc and measured strength increases of over 40 percent. Additional simulation work has predicted similar nanoparticle array defect phases for Al–Zr,^45^ again forming a pearl-necklace pattern that is confined. While not confirmed experimentally to date, atomistic modeling studies^45^ have also predicted platelet-shaped dislocation defect phases in Al–Cu, which resemble Guinier–Preston zones and restructure the dislocation core into faceted segments, and defect phases where the dislocation core is delocalized. An example of this latter type is presented in the bottom right of Figure 5, where segregation to the stacking fault in Cu–Zr results in the formation of a defect phase comprised of stacked planes that resemble the Cu_5_Zr intermetallic phase, yet do not create the full three-dimensional (3D) crystal structure or maintain the exact stoichiometry. Other options for core delocalization likely exist and should be studied. Platelet array defect phases require climb prior to slip^48^ and defect phases with core delocalization require dislocation nucleation,^46^ meaning pronounced mechanical property changes are likely to result from these defect phase types and this topic is an area for future study. While examples of dislocation defect phases are not plentiful in the literature for hcp metals, a few scattered observations do exist. For example, Yang et al.^49^ discovered quasicrystalline nanoscale precipitates along the length of dislocations in Mg–Zn that were accompanied by Zn segregation. In this case, the broken symmetry associated with the dislocation core led to Penrose-like random tiling patterns, which then led to precipitate nucleation.

Dislocation defect phase diagrams provide a tool for predicting defect phase formation so that alloy compositions and/or processing routes can be designed appropriately. For metallic solid solutions, defect phases typically occur in the nearby lattice and therefore appear as modifications of the bulk phase diagram. This contrasts with grain-boundary defect phases, where many options, including ordered grain-boundary segregation patterns or even disordered films have little or only an indirect connection to the lattice structure in the bulk material. For dislocations, reduced solubility limits often appear on defect phase diagrams to signify the local enrichment and transformation.^37^ Turlo and Rupert^43,45^ showed that this modification of the solubility limit can be described by accounting for the free energy change due to the presence of the dislocation’s distortion field. The level of modification was shown to depend on the defect density in the dislocation network by Zhou et al.^39^ It is worth noting that in some cases atomistic details of the phase transformations can be altered by the fact that this transition is occurring and restricted to a nanoscale region. For example, Fe–Ni was shown to follow a transformation pathway where an embryo of a bcc-like B2 phase forms first to provide an environment within which the L10 phase can eventually nucleate, thereby reducing the interfacial penalty between the defect phase and the surrounding lattice.^44^ Mianroodi et al.^50^ were able to capture many of these features in a phase-field model for binary solid solutions, showing that the solute lattice misfit results in a strong elastic contribution to both binodal and spinodal behaviors. Tehranchi et al.^11^ have built metastable defect phase diagrams for Fe–Nb and Mg–Al–Ca solid solutions that are capable of representing the kinetic limitations that can prevent an alloy from reaching equilibrium, showing clearly how defect phase formation can be an intermediate step to bulk precipitation. Such considerations are essential when seeking to find the processing conditions where defect phases formation will take precedence over precipitation of bulk phases.

## Dislocation defect phases in ordered intermetallics

Intermetallic compounds, characterized by their sublattice-ordered structures and distinct stoichiometric ratios, represent a class of materials with significant potential for high-temperature structural and functional applications. Their complex atomic arrangements often lead to properties that differ significantly from those of metallic alloys. Despite recent studies highlighting the role of amorphous shear band formation in accommodating strain beyond the initial stages of plasticity,^51^ dislocations remain fundamental in governing the plastic deformation of intermetallics.^52–56^ The unique site-specific environments around dislocations in intermetallics, attributed to bonding anisotropy and coordination preferences within ordered sublattices, strongly influence solute segregation behaviors, often in ways distinct from those observed in metallic solid solutions. Furthermore, the intrinsic brittleness of many intermetallic compounds, alongside the solute effects that can either facilitate or hinder dislocation motion, underscores the need for a deeper understanding of their deformation mechanisms. As previously discussed, there are often structural elements reminiscent of favorable bulk ordered phases that form at dislocation defect phases in solid solutions, with local variations in structure or composition that differ. It is therefore of interest to elucidate the mechanisms of motion of dislocations in ordered phases not only for their own sake, but also with a view to better understanding dislocation defect phase stability, motion, and properties in metallic alloys. The emerging concept of dislocation defect phases, namely distinct and solute-stabilized dislocation core structures that evolve with temperature and composition, offers a powerful framework for bridging atomic-scale structural transitions with macroscopic mechanical response in these structurally complex crystals.

## Topologically close-packed phases

Topologically close-packed (TCP) phases are a prevalent class of intermetallic compounds known for their complex crystal structures and exceptional high-temperature stability. Among them, Laves phases (i.e., C14, C15, and C36 types) are the most common and extensively studied due to their frequent formation in multicomponent alloys and significant influence on mechanical properties. The most energetically favored basal or {111} slip mechanism in Laves phases is synchroshear, as confirmed by *ab initio* and atomistic simulations,^57,58^ involving the coordinated glide of two coupled Shockley partial dislocations within the basal or {111} triple-layer. Recent atomistic simulations have revealed that synchroshear operates via thermally activated kink-pair nucleation and propagation, with the rate-limiting step being the kink-pair formation.^18^ Importantly, point defects, such as vacancies and antisites, that tend to segregate at dislocation cores, can significantly lower energy barriers and facilitate dislocation motion,^14^ demonstrating how point defect decoration at dislocation cores modulates plasticity and leads to softening in off-stoichiometric compositions in these complex intermetallic systems.^59,60^ Complementing these insights, Šlapáková et al.^61^ provided atomic-resolution evidence of solute-enriched basal planar faults in Nb-rich C14 NbFe_2_ Laves phases. In this system, excess Nb is accommodated not only via point defect decoration, but also by the incorporation of coherent Zr_4_Al_3_-type building blocks as planar faults. These stacking configurations constitute metastable defect phases, characterized by distinct local chemical environments and structural transformations, which truncate nonbasal slip systems and may thereby influence nonbasal dislocation mobility and the overall mechanical response.

In addition to basal slip, nonbasal dislocations, such as prismatic and pyramidal partials, play a key role in accommodating *c*-axis deformation in C14 Laves phases. These nonbasal planar faults can form through different dislocation mechanisms, depending on temperature and deformation conditions. At high temperatures, diffusion-assisted shuffle processes dominate, leading to structurally coherent and ordered faulted regions with site-specific solute occupations and tcp motifs (e.g., Zr_4_Al_3_ and MgCu_2_-type building blocks).^17,62^ In contrast, at low temperatures or under high strain rate deformation, glide-based mechanisms become more prevalent, resulting in stepped and disordered fault structures.^17,62^ Zhang et al.^63^ demonstrated that coherent domain boundaries form in NbCr_2_ Laves phases during compressive deformation and persist even after prolonged annealing at high temperature, indicating their thermal stability. These domain boundaries and stacking faults exhibit distinct atomic arrangements and local chemistries, often involving point defect partitioning, reinforcing the concept of dislocation-induced planar defects as phase-like entities within the defect phase framework. Beyond hexagonal Laves phases, recent work by Peter et al.^64^ revealed that dislocations and twin boundaries in the cubic C15 CaAl_2_ serve as preferred oxygen diffusion pathways, leading to local chemical and structural transformations. Using atomic-resolution electron microscopy and spectroscopy, they identified the formation of metastable dislocation phases, including coherent Al-rich A1 phases and amorphous Al_2_O_3_, driven by a coupled chemomechanical stimulus at the dislocation core.

**Figure 6:**
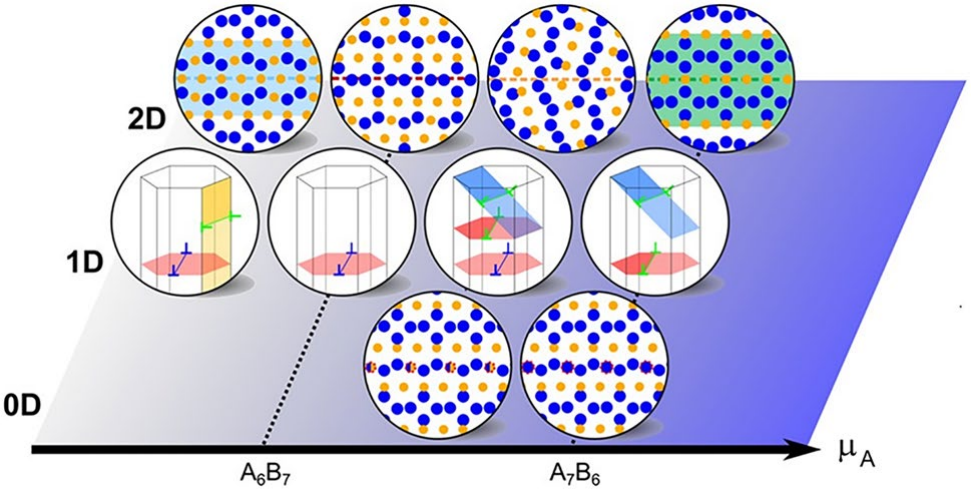
Zero-dimensional (0D) to two-dimensional (2D) defect phases in topologically close-packed µ-phases as a function of the chemical potential of the larger A-atoms. The sublattice order induced by A or B atoms available outside of the ideal stoichiometric compositions controls the prevalence of different dislocations, twins and stacking faults. Reprinted with permission from Reference 66.

**Figure 7:**
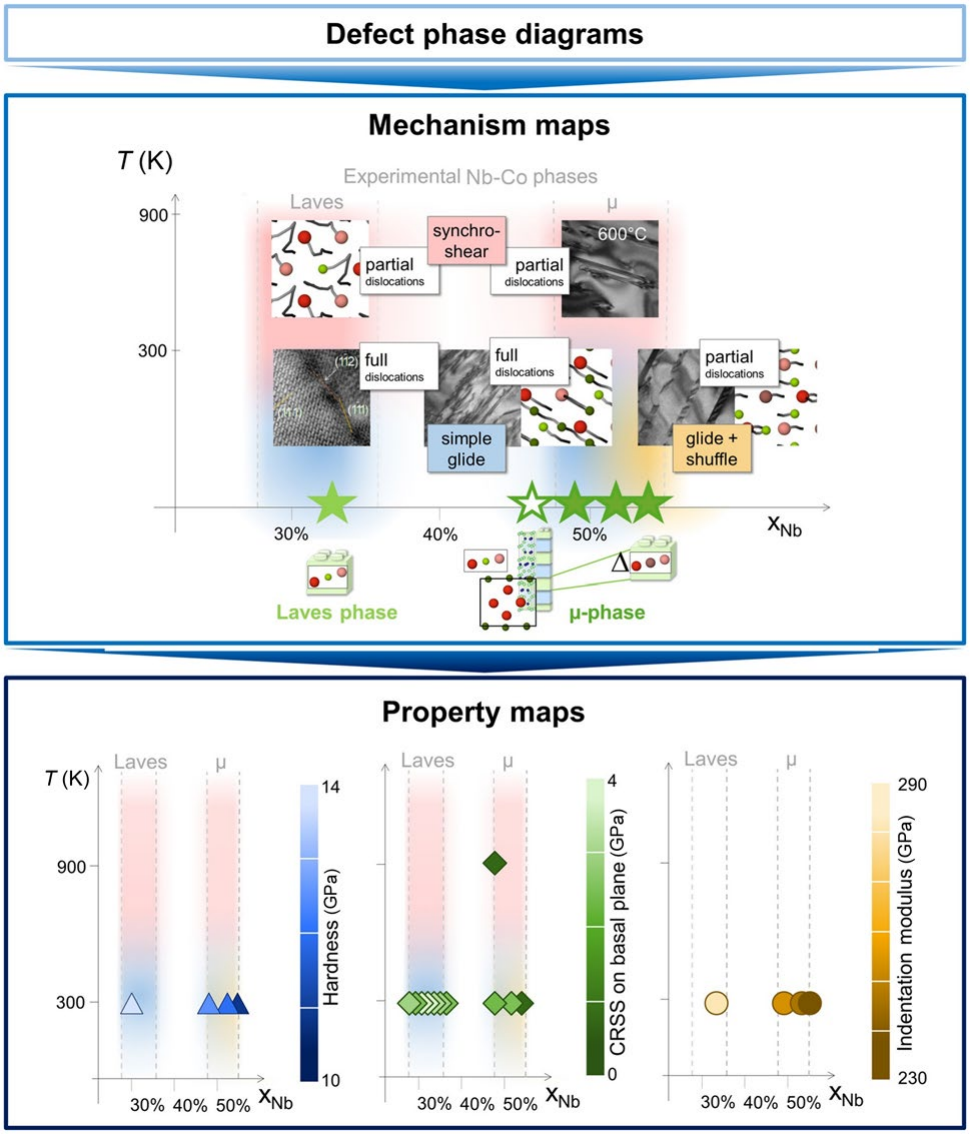
From defect phases to mechanism and property maps: dislocation mechanisms and properties in binary Nb–Co Laves and µ-phases. Data from References 53, 65, and 67.

The µ phase consists of alternating Laves phase (MgCu_2_-type) and Zr_4_Al_3_-type building blocks stacked along the *c*-axis. Recent studies on Co–Nb µ-phases have revealed that varying Nb content alters site occupancies, especially within the Laves triple layers, where Co atoms are increasingly replaced by Nb in Nb-rich compositions.^53,65^ This substitution significantly reduces the basal shear modulus and indentation modulus, as well as decreases hardness and causes a dramatic drop in basal critical resolved shear stress by over 60% from Nb_6_Co_7_ to Nb_7_Co_6_. Deformation in µ phases occurs predominantly via basal slip, either through full or partial dislocation motion, depending on composition and temperature. In Nb-rich compositions or at elevated temperatures, stacking faults mediated by partial dislocations become the dominant deformation mode. Unlike the strongly thermally activated synchroshear-induced mechanisms characteristic of Laves phases, µ phases in Nb-rich off-stoichiometry exhibit a transition to easy glide-and-shuffle slip mechanisms via local atomic rearrangements. These transformations, governed by changes in chemical potential, give rise to distinct defect phases, namely dislocation and fault structures characterized by specific solute distribution and thermodynamic stability (**Figure** 6). Such behavior can be systematically captured in mechanism maps (**Figure** 7), linking local atomic arrangements and site occupancies to dominant slip modes (e.g., full versus partial dislocations, synchroshear versus glide-and-shuffle). These mechanism maps, in turn, can be correlated with macroscopic property maps, such as critical resolved shear stress, hardness, and indentation modulus, providing a predictive framework that connects thermodynamic variables to mechanical performance.

## B2 phases

Studies on B2-ordered FeAl alloys have revealed that point defects play a critical role in modifying dislocation core structures and their mobilities through segregation and defect stabilization. Morris et al.^68^ demonstrated that the well-known anomalous stress rise found at intermediate temperatures in FeAl is due to the pinning of superdislocations by thermal vacancies. These vacancies form dense and stable populations that significantly strengthen the material, analogous to solute hardening. Blavette et al.^69^ used atom probe tomography to directly observe boron-enriched Cottrell atmospheres at edge dislocations in FeAl, showing significant local Al depletion and B enrichment up to 2 at.%, suggesting a strong interaction between dislocations and B solutes. Fraczkiewicz et al.^70^ expanded on this by identifying both equilibrium and nonequilibrium segregation mechanisms of B solutes to grain boundaries and dislocations, with field ion microscopy images revealing rod-like B-rich fingers that follow 〈100〉 dislocations and exhibit distinct Al depletion, evidence of a solute-dislocation complex fault structure. Viguier et al. ^71^ provided a structural characterization of the complex fault in B-doped FeAl alloys, showing that these planar defects, formed by segregation of B–Al vacancy complexes, exhibit mixed stacking fault and antiphase boundary character, with displacement vectors having both in-plane and out-of-plane components. These findings support the emerging concept that dislocations in intermetallics can host thermodynamically distinct, solute or vacancy-stabilized defect phases that fundamentally influence mechanical behavior. Considering these defects in B2 alloys in conjunction with the B2/L10 structures at dislocation defect phases in the Fe–Ni systems described above highlights the potential of a coherent framework allowing a coherent analysis across material systems that are related by their chemistry or the structures they contain.

## Other intermetallic phases

Evidence of defect phase formation and their impact on materials properties is evident also from many works on other intermetallic phases.

Early atom probe tomography studies by Horton and Miller^72^ showed that interstitial boron segregates to dislocations and planar defects in Ni_3_Al, modifying the local chemistry and enhancing ductility through boundary stabilization. Later, Jayaram and Miller^73^ extended this to NiAl, demonstrating that Zr segregates to 〈100〉 dislocations and grain boundaries, forming Zr-rich ribbons and pinning dislocations, which raised the yield stress and the ductile-to-brittle transition temperature, highlighting a tradeoff between strength and ductility due to dislocation pinning. More recently, Wang et al.^74^ employed multiscale quantum mechanics/molecular mechanics simulations to study C and Nb segregation at screw dislocations in γ-TiAl. They found that solute atoms at the dislocation core not only alter core energy, but can induce dislocation core recombination, violating Frank’s rule and promoting dislocation mobility. This solute-driven restructuring reduces critical resolved shear stress and enhances ductility, suggesting an atomistic mechanism by which solute-dislocation interactions can actively transform defect structure to improve mechanical performance.

Smith et al.^75,76^ demonstrated that solute segregation and local phase transformations along stacking faults in Ni-based superalloys act as planar defect phases that significantly influence high-temperature creep behavior. In ME501, segregation of Nb, Ta, and Ti drives a stress-induced transformation from γ^*′*^ to a nanoscale Z-phase along superlattice extrinsic stacking faults, which raises the energy barrier for nanotwin formation and enhances creep resistance.^75^ They also compared ME3 and LSHR alloys, finding that ME3 forms a disordered γ-like phase along faults, while LSHR forms ordered Co_3_W (χ-phase) and possibly η-phase, inhibiting fault propagation and improving creep strength.^76^ These findings establish dislocation-associated local structural transitions as stress-stabilized defect phases critical to alloy design.

For diamond cubic GeSn, Mukherjee et al. ^77^ investigated solute segregation at dislocations in strained, metastable layers using atom probe tomography. They revealed that dislocations act as preferential sites for Sn segregation, forming ”Sn pipes” with concentrations up to four times the matrix value. In early annealing stages, Sn atoms migrate toward dislocations, forming nanoscale random atomic clusters, which serve as precursors to phase separation. Prolonged annealing activates pipe diffusion along dislocation cores, transporting Sn atoms to the surface and completing the segregation process. This study demonstrates how dislocation-solute interactions drive localized phase transformation in semiconductors, reinforcing the concept of defect phases as thermodynamic entities evolving over time.

## Perspective for materials design

**Figure 8:**
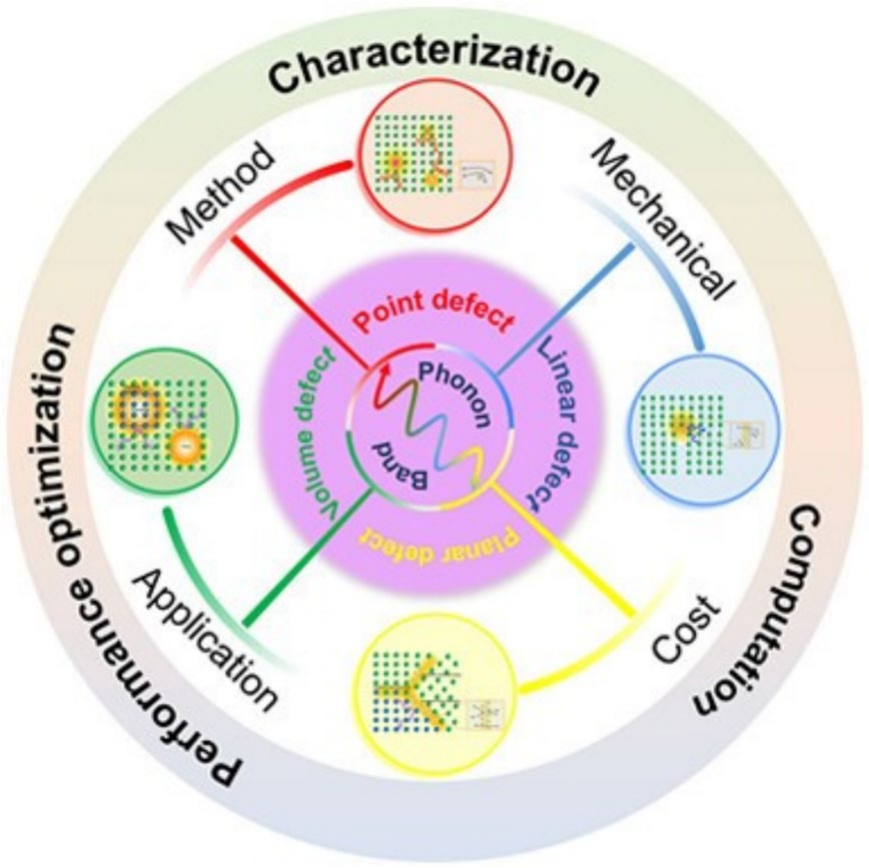
Perspectives of defect engineering in thermoelectrics. Adapted with permission from Reference 80. ©t 2024 American Chemical Society.

There is now ample experimental and computational evidence that defect phases exist not only for grain boundaries, but also for other defect dimensionalities and structures, such as point defects, planar faults, and in particular for dislocations, as previously outlined. This article has largely focused on mechanical properties with dislocations their main carrier facilitating plastic deformation. However, dislocations also play a vital role where functional properties are concerned and we expect that the formation of defect phases will affect materials’ functions well beyond the mobility of dislocations. Dislocations have already been explored in many materials and the interaction of lattice defects of all dimensionalities is not unique to the mechanical properties of structural materials. Examples of dislocations affecting materials performance in functional materials ranges from the relief of coherency strains in semiconductor multilayers^78^ over tailoring of electrical conductivity, superconductivity, photoconductivity, or thermal conductivity in ceramics^79^ to efficiency of thermoelectrics^80^ (**Figure** 8). Dislocations introduce elastic strain fields and localized core distortions that serve as effective phonon scattering centers, significantly reducing lattice thermal conductivity without substantially compromising electrical transport.^81^ Dislocations have emerged as critical design elements for enhancing the thermoelectric figure of merit, particularly through the tuning of dislocation density via strategies such as vacancy engineering or thermal processing. Solute segregation around dislocation cores, particularly the formation of Cottrell atmospheres, can further stabilize dislocation structures and modulate local electronic environments, thereby amplifying phonon-scattering effects and contributing to overall thermoelectric performance gains.^82^ Engineering the defect microenvironment in materials with strong electrical charge and magnetic spin interactions could also facilitate the manipulation of functional properties, such as ion transport along charged extended defects,^83^ magnetoplastic coupling in ordered systems, or light and field-responsive defect motion.^84–86^

Owing to the fundamental work still needed to explore dislocation defect phases, we have also refrained from expanding on the data and knowledge already available with respect to their interactions beyond the role of the presence of point defects in controlling dislocation motion.^18^ The role of dislocations and disconnections in grain boundaries is analogous, but also even more complex even without considering a locally constrained atomic structure and chemistry at the grain boundary or the dislocations and disconnections it may contain.^87^ With Pemma et al.’s^88^ work on the motion of grain-boundary defect phases and the role of their atomic structure with respect to disconnections is beginning to emerge as well. Other articles in this issue review grain-boundary defect phases and their stability, structure, mobility, and impact on microstructure evolution as well as functional properties.^89^

As is frequently the case when exciting new avenues for exploring and understanding fundamental concepts in materials physics and design arise, they trigger new questions. In the next years, we expect that explorations of dislocation defect phases will revolve around more than finding, predicting, and connecting dislocation defect phases to materials properties. The next frontiers in leveraging this knowledge and the thermodynamic frameworks that are being developed will likely center around several challenges and opportunities. We identify several of these, as well as fundamental questions, to guide future exploration:

- **Connecting more defect phases and their transitions with mechanisms and properties.** Much as the grain-boundary defect phase community has revisited conventional wisdom on phenomena such as grain growth, grain-boundary embrittlement, activated sintering, and heterogeneous phase nucleation in the context of the defect phase framework, a large opportunity space exists to rethink the multitude of properties governed by dislocations and point defects by unifying defects with phase equilibria. However, the detailed and meticulous experimental characterization that is required for compelling demonstrations of causal relationships between defect phases and properties at 1D and 0D defects will demand state-of-the-art tools and models. *Question: How can we design for structures/orderings that are not predicted by bulk phase diagrams?*
- **Expanding these insights beyond the thermodynamic competition of defect phases for solutes in a matrix to include their interactions.** In most cases, the segregation of solutes is only the first stage in establishing the chemical conditions that promote a defect phase transition. Materials design strategies that make use of the defect phase framework will have to consider the kinetics of the available solute reservoir in addition to the rich interactions that these species will have at the defect core and their environment (e.g., the strain field of a dislocation). Enumerating the interactions in both dilute and concentrated defect states and across the number of defect characters in multicomponent systems is a grand challenge in predicting the range of property outcomes. The intrinsic transient nature of such states in mobile defects coupled with rapid transport channels (e.g., pipe diffusion along dislocations) introduces additional complexities to be tackled. *Question: What is the tradeoff between defect phase tunability and defect mobility?*
- **Broadening the description of individual defect phases to interacting networks of defect phases.** A natural hierarchy exists among defects, with higher dimensional defects (e.g., 2D grain boundaries) comprising a network of lower dimensional defects (e.g., 1D disconnections within a grain boundary) that control the defect structure and properties. Hosting defect phases across this hierarchy will likely imply an interacting network that self-organizes and responds to stimuli in novel ways. *Question: How can we distinguish defect phases that emerge from defect core interactions and those from longer-range strain fields that (tensorially) modify known thermodynamic transitions?*

- **Providing a coherent framework that allows multiphysics connections between defect phases and properties, whether they are structural/mechanical or functional in nature to enable multiphysics materials design**. A holistic framework in this vein incorporating all degrees of freedom (e.g., chemical, charge, magnetic spin, and structural) as possible order parameters would enable the prediction of localized phase evolution, transport, and the multiphysics coupling between several fields. One can envision a rich number of emergent phenomena that could be hosted, particularly in ordered systems (such as the intermetallics previously mentioned) with multiple sublattices, with implications for optimizing coupled property suites (e.g., mechanical/corrosion/radiation resistance). Models that treat all of these contributors to a material’s free energy and can handle the mesoscale nature of extended defects are needed. *Question: How do additional degrees of freedom at the defect compete or assist in the progression of defect phase transitions?*
- **Leveraging this knowledge to predict materials properties not yet explored through experiments and computations**. The establishment of defect-phase-property diagrams to complement defect phase diagrams would help guide materials design strategies. Useful graphical tools and mapping approaches reminiscent of Ashby-type property diagrams could help identify ”white spaces” that could be filled by designing for appropriate defect phase content. Theoretical frameworks following the above could also define bounds on properties governed by defects. *Question: What are the bounds on properties governed by defect phases?*

## Conclusion

Defect phases are a fundamental aspect of materials science that significantly influence materials properties and microstructural evolution. By recognizing defects as thermodynamically metastable defect phases and constructing defect phase diagrams, a powerful tool for predicting and controlling material behavior is emerging.

Understanding the interplay of defect phases across different dimensionalities — point defects, dislocations, stacking faults, grain and phase boundaries — enables a comprehensive approach to materials design. This unified framework holds the potential to link atomistic complexity with macroscopic properties, facilitating a paradigm shift in the description and engineering of advanced materials.

Focusing on dislocations as the defects that unite a high multiplicity of potential defect phases with a high mobility giving rise to easily accessible materials properties, we showcase that evidence of their existence and potential for materials design is ubiquitous. Studies across metals, ordered intermetallics, and ceramics, even where they are not performed with the systematics of defect phase diagrams in mind, reveal that by tailoring dislocations as well as their interaction with other defects, mechanical and functional materials can be tailored as well.

However, just as the potential of understanding these defect phases emerges, new questions arise. Answering these fundamental unknowns, from the dynamics and kinetics of dislocation defect phases over the interplay of the low energy defect states and the low energy ordered structures present or not in the bulk phase diagrams to the scope in which not only structure and chemistry, but also local atomic bonding can be changed locally, will expand our understanding of defect stability and competition. In addition, our understanding of the fundamental materials physics of defects will expand and improve, as will our predictions for materials that may exhibit an exceptional range of properties accessible through purposeful tuning of defects of all dimensionalities.
